# Supplementary material for: Somatic deficiency of the human E3 ubiquitin ligase CBL in leukocytes impairs B cell but not T cell development and function
Source: Nat Immunol. 2026 Jan 15;27(2):308–22. doi: 10.1038/s41590-025-02381-7 (PMC12864045; doi:10.1038/s41590-025-02381-7)
Supplement: Supplementary file 1 — Supplementary Information [file 41590_2025_2381_MOESM1_ESM.pdf]

nature immunology

Supplementary information https://doi.org/10.1038/s41590-025-02381-7

Somatic deficiency of the human E3 ubiquitin ligase CBL in leukocytes impairs B cell but not T cell development and function

In the format provided by the authors and unedited

Supplemental Material and Methods, Vatovec et al. 2025:

Case Reports

Patients P1-P9 have previously been reported in Bohlen et al 2024, JCI. For these patients we focus here on the description of the infectious episodes. We include more indepth reports for the newly described patients P9 and P10.

CBL-LOH patients have severe infectious diseases

P1, P2 and P3 are monozygotic triplets, 13 years of age at the time of study and born to non-consanguineous French parents. All three male patients experienced several episodes of pneumonia or pulmonary infections, between the ages of 1 to 6 years. While Streptoococcus and Hemophilus species were detected in bronchoalveolar lavage fluid, their contribution to disease was not clearly established. Computed tomography (CT) scans were suggestive of bacterial origin of disease (Figure S1A). After remission of pneumonia, CT of their lungs revealed distributed parenchymal lesions at 9 years old (Figure S1B). P4 is a 26-year-old Italian woman who suffered from life-threatening pneumonia with septic shock at age 4 years. Several autoinflammatory and autoimmune manifestations were also diagnosed (details in (24)). P4 had another severe infectious episode at 22 years of age, when she was under corticosteroid. P5, 4 years old at the time of study, had pneumococcal encephalitis with sepsis at age 4 months and later developed several severe urinary tract infections (25). She later developed juvenile monomyelocytic leukemia (JMML), which resolved spontaneously. P6 was asymptomatic with moderate splenomegaly during childhood and is 19 years at the time of this study. P7 had pneumonia when she was 15 months old, with thorax radiography suggesting a bacterial cause of infection (Figure S1C), which was empirically and successfully treated with ampicillin. At this time, she tested positive for CMV by serology and PCR (1080 counts/µl by PCR), which resolved without antiviral treatment. P8 had JMML without striking infectious events. P9 had JMML and severe vasculitis, but without notable infectious episodes. Testing of patients at ages 3 - 26 years indicated intact antibody (Ab) responses to childhood infections and vaccines, as shown by the detection of Abs against various microbes using clinical serology testing and virome-wide serological profiling at similar levels to healthy, age-matched pediatric donors (Figure S2A-B). Thus, homozygosity for CBL UbLOF variants in blood cells are associated with unusually severe infections, particularly those caused by bacteria.

Patient P10:

P10 was a male infant of Iranian origin who developed severe infections early in life, first presenting at one month with critical SARS-CoV-2 pneumonia requiring prolonged intensive care. From four months of age he developed complications of BCG vaccination, including recurrent right axillary suppurative lymphadenitis that persisted despite repeated drainage and ultimately required excision, where pathology confirmed necrotizing granulomatous inflammation and cultures were positive for acid-fast bacilli. He also developed progressive splenomegaly without abscess formation, and bone marrow examination showed monocytosis with myeloblasts suggestive of a JMML-like picture, though cultures were negative for mycobacteria. The patient was treated with multidrug antimycobacterial therapy and adjuvant interferon-γ, with partial clinical stabilization. However, at 13 months he presented again with fever, pallor, and respiratory distress, alongside massive splenomegaly, severe cytopenias, and coagulopathy. Despite intensive supportive care, he developed multi-organ failure and died within 5 days, without evidence of ongoing viral or mycobacterial infection at the time of death.

Patient P11:

P11 was a 6-year-old Indian boy with recurrent and severe infections beginning at 3 years of age, characterized by persistent fever, pneumonia with bilateral lung consolidations, multiple skin and soft tissue infections (facial and peri-orbital abscesses, tinea corporis), and massive splenomegaly with hepatomegaly. Despite extensive evaluation, no pathogen could be consistently isolated from his pulmonary disease, but recurrent staphylococcal skin abscesses and fungal lesions were identified. Hematologic investigations revealed evolving cytopenias with leukoerythroblastosis, raising initial concern for juvenile myelomonocytic leukemia, although the clinical course was dominated by infection and inflammatory features, including markedly elevated ESR and hypergammaglobulinemia (IgG 2400 mg/dL, IgA 275 mg/dL). The child deteriorated with progressive hypersplenism and pulmonary complications, culminating in fatal infection-related decompensation at age 6, after which a homozygous private variant in CBL (p.Cys416Arg) was identified on genetic testing.

Extended Data Figure Legends:

Extended Data Figure 1: Clinical Imaging of patients' torso. **(a)** CT scan during pneumonia of P1 at 5 years of age. **(b)** CT scans of parenchymal lesions of P1, P2 and P3 at 9

years of age. **(c)** Pneumonia in X-ray imagine of P7 at 15 months old. **(d)** X-ray image of P10 at 7 months old with lymphadenopathy affecting the right axillary lymph node.

Extended Data Figure 2: (a) Serology for the indicated microbes of CBL-LOH patients, their relatives and healthy donors. The serology is indicated as the $\log_2$(fold-change) over the positivity limit. **(b)** Virome-wide serological profiling (virscan) of CBL-LOH patients and healthy donors. Signals indicate the adjusted species score calculated from all peptide counts of the indicated species.

Extended Data Figure 3: Characterization and functional study of patients NK and T cells. **(a)** CytoF immunophenotyping of patients total $CD3^+$ T cells, NK cells and indicated NK cell subsets. Controls 0-3 y.o. n = 2, Controls 4-15 y.o. n = 9, Controls 16-100 y.o. n= 28, pediatric patients (LOH) n = 5, adult patients (LOH) n = 2, heterozygous individuals n = 3, mean ± s.d. Statistical significance was assessed with multiple Mann Whitney tests adjusted for multiple testing. *$p<0.05$. **(b)** Assessment of T cell proliferation of patients P1, P2 and P3. (top) gating strategy, (bottom) CFSE dilution plots. **(c)** Intracellular TNF production of $CD4^+$ and $CD8^+$ T cell blasts in homozygous (n = 4), heterozygous (n = 2) and wildtype (n = 5) state of *CBL* Ub^{LOF} variants. Line indicates the mean of the displayed datapoints. **(d,e)** Intracellular and extracellular cytokine production by patients T_{fh} cells stimulated with CD2/CD3/CD28 under T_h0 conditions for 5 days. **(d)** Intracellular staining of the indicated cytokines. Healthy donors (n = 17), CBL-LOH patients (n = 4). Mann-Whitney testing with correction for multiple testing did not reveal significant ($p< 0.05$) differences between healthy donors and CBL-LOH patients. Line indicates the mean of the displayed datapoints. **(e)** Extracellular detection of cytokines by ELISA. Healthy donors (n = 6), CBL-LOH patients (n = 3). Mann-Whitney testing with correction for multiple testing did not reveal significant ($p< 0.05$) differences

between healthy donors and CBL-LOH patients. Line indicates the mean of the displayed datapoints.

Extended Data Figure 4: (a) Quantification of B-cell subsets in cryopreserved PBMCs from healthy donors, heterozygous carriers, and CBL-LOH patients of the indicated ages by mass cytometry. Controls 0–3 y.o. n = 2; controls 4–15 y.o. n = 9; controls 16–100 y.o. n = 28; pediatric CBL-LOH n = 5; adult CBL-LOH n = 2; heterozygous individuals n = 3. Mean ± s.d. Statistical significance was assessed using two-sided Mann–Whitney tests with correction for multiple testing. **(b)** Frequency of B-cell subsets in healthy donors (n = 10) and CBL-LOH patients (n = 4) by flow cytometry. Mean ± s.d. Significance was evaluated with two-sided Mann–Whitney tests adjusted for multiple comparisons. **$p < 0.005$. **(c)** MFI of B-cell markers on CD21^{lo} and transitional B cells from healthy donors (n = 6) and patients P1–3 (n = 3). Lines show means. **(d–f)** Modeling the CBL ΔExon8 variant in primary human CD34^{+} HSPCs. **(d)** Agarose gel electrophoresis of PCR products from AAVS1 or CBL loci 72 h after nucleofection; representative of >5 biological replicates. **(e)** Editing efficiency using sgRNA pair 1+2 by NGS, showing ~80% of the ~400 bp exon-8 deletion. **(f)** Western blot of CBL protein levels after CBL or AAVS1 editing. **(g)** Quantification of three biological replicates from (f). Mean ± s.d. **(h)** NGS quantification of exon-8 deletions for all three guide pairs (n = 2 biological replicates). Bars show means. **(i)** Editing efficiencies at days 8 and 21 in differentiation cultures by NGS. Mean ± s.d. from three biological replicates. **(j–m)** Bulk RNA-seq of AAVS1- and CBL-edited CD19^{+}CD10^{+}CD20^{low} HSPC-derived B-cell progenitors. **(j)** Gene-set enrichment analysis showing significantly enriched (red) or depleted (blue) pathways (NES: normalized enrichment score). **(k,l)** Differential expression of leading-edge genes in the **(k)** Hallmark G2–M checkpoint and **(l)** Hallmark mTORC1 signaling gene sets (Z-transformed normalized counts). **(m)** Transcriptional overlap between CBL-edited and PI3K^{GOF}

progenitors; Venn diagrams show shared significantly up- or downregulated genes, with overlap significance by binomial test.

(a) Quantification of the indicated B cell subsets in the peripheral cryopreserved mononuclear cell blood of healthy donors, heterozygous healthy donors and CBL-LOH patients of the indicated ages as determined by mass cytometry. Controls 0-3 y.o. n = 2, Controls 4-15 y.o. n = 9, Controls 16-100 y.o. n= 28, pediatric patients (LOH) n = 5, adult patients (LOH) n = 2, heterozygous individuals n = 3, mean ± s.d. The statistical significance of differences was assessed in multiple two-sided Mann-Whitney tests, with correction for multiple testing. Mean ± s.d. Statistical significance was assessed with multiple Mann Whitney tests adjusted for multiple testing. (**b**) Frequency of B cells subsets in healthy donors (n = 10) and CBL-LOH patients (n = 4) cryopreserved PBMCs as determined by flow-cytometry. Mean ± s.d. The statistical significance of differences was assessed in multiple two-sided Mann-Whitney tests, with correction for multiple testing. **p<0.005. **(c)** Intensity (MFI) of B cell markers on $CD21^{lo}$ and transitional B cells of heathy donor (n= 6) and patients P1-3 (n = 3). Line indicates the mean of the displayed datapoints. **(d-f)** Modelling CBL ΔExon 8 variant in primary human CD34+ HSPCs. **(d)** Agarose gel electrophoresis of PCR products for the *AAVS1* or *CBL* loci performed on genomic DNA isolated from CD34+ HSPCs 72 hours after nucleofection. Representative of *n* > 5 biological replicates. **(e)** Editing efficiency at the *CBL* locus with sgRNA pair 1+2 by NGS, showing ~80% editing efficiency of ~400bp deletion containing exon 8. **(f)** Western blot showing CBL protein levels after editing of the CBL or AAVS1 locus. **(g)** Quantification of three biological replicates of this western blot. Mean ± s.d. **(h)** Quantification of exon 8 deletions at the *CBL* locus determined by NGS for all three guide pairs. *n* = 2 biological replicates. Bar shows the mean. **(i)** Editing efficiencies on day 8 and day

21 post editing in differentiation cultures as determined by NGS. Mean ± s.d. of three biological replicates **(j-m)** Bulk RNA-seq on AAVS1- and CBL-edited $CD19^{+}CD10^{+}CD20^{low}$ HSPC-derived B progenitor cells. **(j)** Gene set enrichment analysis with pathways for which significant enrichment (red) and depletion (blue) was detected. **(k,l)** Differential gene expression between control AAVS1- and CBL-edited B-lineage cells for leading edge genes in **(k)** Hallmark G2-M checkpoint and **(l)** Hallmark mTORC1 signaling gene sets. Colors reflect Z-transformed normalized read counts. NES: Normalized enrichment score. **(m)** Transcriptional overlap between CBL-edited and $PI3K^{GOF}$ HSPC-derived B cell progenitors. Venn diagrams showing overlap between significantly differentially expressed genes in $PI3K^{GOF}$ and CBL-edited samples. Left: genes upregulated in both $PI3K^{GOF}$ and CBL Ub^{LOF} samples as compared to $AAVS1^{KO}$. Right: genes downregulated in both comparisons. Overlap significance was calculated using a binomial test.

Extended Data Figure 5: Intact cell extrinsic determinants of B cell development and function. **(a)** Levels of sCD40L, APRIL and BAFF in the plasma of CBL-LOH patients (n = 6) and healthy donors (n = 14). Line shows the mean of the displayed datapoints (one point per individual assessed). **(b)** Quantification of the production of sCD40L, APRIL and BAFF by PBMCs of CBL-LOH patients (n = 3) and healthy donors (n = 5). Line shows the mean of the displayed datapoints (one point per individual assessed). **(c-f)** Impact of sCD40L levels on B cell differentiation in vitro using CD34+ HSPCs. **(c,d)** B cell output at day 21 of co-culture of CD34+ HSPCs from two healthy donors. The MS5 co-culture was supplement with IL-7 (20 ng/mL) and the indicated doses of sCD40L. Mean of dots that represent technical replicates. **(e,f)** Quantification of B cell subsets based on CD10 and CD20 marker expression. Cells were treated as in **(c,d)**. Mean ± s.d. **(g)** IL-21 production by sorted $CD4^{+}$ naïve and memory T cells upon the indicated stimuli. Mean ± s.d. **(h)** Surface staining of CD38 expressed on CD19+

HSPC-derived B cell progenitors edited with the indicated sgRNAs. Mean ± s.d. of three biological replicates. **(i)** Surface staining of CD38 expressed on HEK293T, REH and BJAB cells as determined by flow cytometry. Mean ± s.d. of three biological replicates. **(j)** CBL Y371C KI BJAB (n = 9) cells are not more resistant than WT (n= 5) cells to BCR-induced apoptosis. Mean ± s.d. of clones over three independent experiments.

Extended Data Figure 6: (a-f) Western blots of wildtype and CBL Y371C KI REH cells upon CD38 crosslinking with daratumumab for the indicated time periods (min/h). **(b,c, e,f)** Shows quantifications of three biological replicates. Mean ± s.d. Statistical significance was assessed with Mann Whitney tests. *p<0.05. **(g,h)** ERK phosphorylation upon CD38 crosslinking in gene-edited HSPC-derived CD19+ B progenitors. Total CD19+ **(g)** or CD19+CD20high **(h)** B progenitors were sorted from co-cultures edited at the AAVS1 or CBL locus. Cells were stimulated for 15 minutes with Daratumumab, followed by fixation, permeabilization and intracellular staining for phosphorylated ERK (pERK). n = 2 biological replicates. Bars show the mean.

Extended Data Figure 7: (a,b) Transitional B cells (n=4 independent experiments) and naïve B cells (n=12 independent experiments) were sort-purified from PBMCs of healthy donors and then cultured in vitro. Levels of IgM, IgG and IgA were measured in supernatants from transitional and naïve B cells after 7 days following stimulation with: **(a)** CD40L alone or in combination with CpG and/or BCR crosslinking, or **(b)** CD40L and IL-21. Box and whiskers indicate median (central line), quartiles (box) and deciles (whiskers). **(c)** Western blot of CBL KO and stable lentiviral overexpression on BJAB cells. **(d)** Sanger genotyping of BJAB CBL Y371C KI and control wildtype cells. **(e)** Ig production of control wildtype and CBL Y371C KI BJAB cell lines within 24 hours of culture unstimulated (n = 4 biological

replicates) and upon IL-4 stimulation (n = 3 biological replicates). Mean ± s.d. The statistical significance of differences was assessed in multiple two-sided Mann-Whitney tests, with correction for multiple testing. *p<0.05. **(f)** Quantification of three biological replicates of the western blot shown in Figure 5E. Mean ± s.d. Statistical significance was assessed with Mann Whitney tests. *p<0.05. **(g)** Pathway enrichment analysis of bulk RNA-sequencing of healthy donor and CBL LOH patients' primary naïve B cells directly after sorting from cryopreserved PBMCs or after 24 hours of non-stimulated culture. NES: normalized enrichment ratio. p values were adjusted for multiple testing. **(h)** Immunoglobulin production by sorted primary B cell subsets of healthy donors (n = 10) and *CBL*-LOH patients (n = 5) from fresh blood samples without stimulation. Supernatants were collected after 24 hours and Igs were measured by ELISA. Line indicates the mean of the displayed datapoints (one point per individual tested). Statistical significance was assessed using Mann Whitney tests and correction for multiple testing. *p < 0.05, ***p< 0.0005.

Extended Data Figure 8: (a) Flow cytometry for Spike tetramers on primary B cells of healthy donors and patient P2. **(b)** Cell painting of healthy donor transitional and memory B cells by the indicated plasma samples. **(c)** Validation of presence of anti-TXLNB autoantibodies in three CBL-deficient patients by multiplex bead assay. Line indicates the mean of the displayed datapoints (one point per individual tested).
