# Supplementary material for: Somatic deficiency of the human E3 ubiquitin ligase CBL in leukocytes impairs B cell but not T cell development and function
Source: Nat Immunol. 2026 Jan 15;27(2):308–22. doi: 10.1038/s41590-025-02381-7 (PMC12864045; doi:10.1038/s41590-025-02381-7)
Supplement: Supplementary file 2 — Reporting Summary [file 41590_2025_2381_MOESM2_ESM.pdf]

nature portfolio

Corresponding author(s): Jonathan Bohlen, Stuart G Tangye

Last updated by author(s): 2025.11.20

Reporting Summary

Nature Portfolio wishes to improve the reproducibility of the work that we publish. This form provides structure for consistency and transparency in reporting. For further information on Nature Portfolio policies, see our Editorial Policies and the Editorial Policy Checklist.

Statistics

For all statistical analyses, confirm that the following items are present in the figure legend, table legend, main text, or Methods section.

n/a	Confirmed	
☐	☒	The exact sample size (*n*) for each experimental group/condition, given as a discrete number and unit of measurement
☐	☒	A statement on whether measurements were taken from distinct samples or whether the same sample was measured repeatedly
☐	☒	The statistical test(s) used AND whether they are one- or two-sided<br>*Only common tests should be described solely by name; describe more complex techniques in the Methods section.*
☒	☐	A description of all covariates tested
☒	☐	A description of any assumptions or corrections, such as tests of normality and adjustment for multiple comparisons
☐	☒	A full description of the statistical parameters including central tendency (e.g. means) or other basic estimates (e.g. regression coefficient) AND variation (e.g. standard deviation) or associated estimates of uncertainty (e.g. confidence intervals)
☐	☒	For null hypothesis testing, the test statistic (e.g. *F*, *t*, *r*) with confidence intervals, effect sizes, degrees of freedom and *P* value noted<br>*Give P values as exact values whenever suitable.*
☒	☐	For Bayesian analysis, information on the choice of priors and Markov chain Monte Carlo settings
☒	☐	For hierarchical and complex designs, identification of the appropriate level for tests and full reporting of outcomes
☒	☐	Estimates of effect sizes (e.g. Cohen's *d*, Pearson's *r*), indicating how they were calculated

Our web collection on statistics for biologists contains articles on many of the points above.

Software and code

Policy information about availability of computer code

Data collection	No applicable.
Data analysis	Graphpad Prism, Flow Jo, Microsoft Excel

For manuscripts utilizing custom algorithms or software that are central to the research but not yet described in published literature, software must be made available to editors and reviewers. We strongly encourage code deposition in a community repository (e.g. GitHub). See the Nature Portfolio guidelines for submitting code & software for further information.

Data

Policy information about availability of data

All manuscripts must include a data availability statement. This statement should provide the following information, where applicable:

- Accession codes, unique identifiers, or web links for publicly available datasets
- A description of any restrictions on data availability
- For clinical datasets or third party data, please ensure that the statement adheres to our policy

All raw data for bulk RNA-seq on primary naïve B cells (GSE307942) and HSPC-derived B cell precursors (GSE307131) have been deposited in the GEO repository and will be made available as of publication date. BCR-sequencing data have been deposited on SRA (PRJNA1328925) and are available as of publication. All raw

data and resources will be made available upon request to the corresponding authors.
There is no original code generated in this study.

Research involving human participants, their data, or biological material

Policy information about studies with human participants or human data. See also policy information about sex, gender (identity/presentation), and sexual orientation and race, ethnicity and racism.

Reporting on sex and gender	This cohort includes both female and male patients, with no distinct phenotype segregation based on sex. Healthy controls of both sexes were also recruited, and no significant differences related to sex were observed.
Reporting on race, ethnicity, or other socially relevant groupings	No information regarding race or ethnicity was collected or analyzed.
Population characteristics	Not applicable. This studies recruitment is based on the presence of rare genetic variants and the size of the cohort is limited.
Recruitment	Patients were recruited due to presence of deleterious variants in the CBL gene by clinicans involved in the CBL consortium.
Ethics oversight	Informed consent was obtained from patients in their respective countries of residence (Iran, Italy, France, Spain, and Germany) in accordance with local regulations and with approval from the relevant institutional review boards (IRB). Physicians responsible for patient care completed a comprehensive questionnaire that recorded demographic data, clinical features, and biological and microbiological findings. No information regarding patients' gender or socioeconomic status was collected. Experiments were carried out in Australia, France, Germany, Sweden, and the United States of America, adhering to local regulations and with IRB approval from Rockefeller University (protocol no. JCA-0699) and INSERM (protocol no. C10-07 and C10-16) for the United States and France, respectively. Healthy donors were recruited from France, Spain, Italy, the United States of America, and Australia. This study was approved by the Sydney Local Health District RPAH Zone Human Research Ethics Committee and Research Governance Office, Royal Prince Alfred Hospital, Camperdown, NSW, Australia (Protocol X16-0210/LNR/16/RPAH/257).

Note that full information on the approval of the study protocol must also be provided in the manuscript.

Field-specific reporting

Please select the one below that is the best fit for your research. If you are not sure, read the appropriate sections before making your selection.

☒ Life sciences ☐ Behavioural & social sciences ☐ Ecological, evolutionary & environmental sciences

For a reference copy of the document with all sections, see nature.com/documents/nr-reporting-summary-flat.pdf

Life sciences study design

All studies must disclose on these points even when the disclosure is negative.

Sample size	The cohort comprises 11 patients, which is a relatively large cohort for such a rare genetic disease. In each experiment and when possible, at least three,but usually more patients were included.
Data exclusions	None
Replication	Experiments were reproduced in typically three biological replicates. Assessment of different patients with the same genotype was interpreted as biological replicates.
Randomization	Randomization was not feasible in a study of rare genetic disease like this.
Blinding	Blinding was carried out when possible (for example when collaborators analyzed BCR-seq data. Often, blinding was not feasible as researchers were working with primary material from patients, blinding of these samples in a clinical research context was not feasible.

Reporting for specific materials, systems and methods

We require information from authors about some types of materials, experimental systems and methods used in many studies. Here, indicate whether each material, system or method listed is relevant to your study. If you are not sure if a list item applies to your research, read the appropriate section before selecting a response.

Materials & experimental systems

n/a	Involved in the study	
☐	☒	Antibodies
☐	☒	Eukaryotic cell lines
☒	☐	Palaeontology and archaeology
☒	☐	Animals and other organisms
☐	☒	Clinical data
☒	☐	Dual use research of concern
☒	☐	Plants

Methods

n/a	Involved in the study	
☒	☐	ChIP-seq
☐	☒	Flow cytometry
☒	☐	MRI-based neuroimaging

Antibodies

Antibodies used: Antibodies used in the study are listed in the methods section of the manuscript, including their references, dilutions etc.

Validation: Antibodies used were typically well validated antibodies in the field. CBL antibody was validated by western blotting on a CBL knockout cell line.

Eukaryotic cell lines

Policy information about cell lines and Sex and Gender in Research

Cell line source(s): Cell lines were obtained from National collections like the ATCC HEK293T and REH) and DSMZ (BJAB).

Authentication: Authenticated cell lines were obtained directly form cell banks. The different laboratories that participated in this research use different commercial services for cell line authentication (e.g. Eurofinsgenomics), the cell lines used here were obtained recently from the cell banks and therefore not specifically re-authenticated when this research was performed.

Mycoplasma contamination: Presence of mycoplasma was regularly assessed by enzymatic and qPCR assays in house. All cell lines used in the study tested negative for mycoplasma at the time of the experiments.

Commonly misidentified lines (See ICLAC register): HEK293T cells for lentivirus production and CD38 surface staining.

Clinical data

Policy information about clinical studies

All manuscripts should comply with the ICMJE guidelines for publication of clinical research and a completed CONSORT checklist must be included with all submissions.

Clinical trial registration: Not applicable

Study protocol: JCA-0699, C10-07 and C10-16

Data collection: See above.

Outcomes: Not applicable.

Plants

Seed stocks: None

Novel plant genotypes: None

Authentication: None

Flow Cytometry

Plots

Confirm that:

☒ The axis labels state the marker and fluorochrome used (e.g. CD4-FITC).

☒ The axis scales are clearly visible. Include numbers along axes only for bottom left plot of group (a 'group' is an analysis of identical markers).

☒ All plots are contour plots with outliers or pseudocolor plots.

☒ A numerical value for number of cells or percentage (with statistics) is provided.

Methodology

Sample preparation	All described in the methods section of the manuscript.
Instrument	BD Fortessa, Aria II, Novocyte
Software	Flow Jo
Cell population abundance	Indicated in the figures and methods section
Gating strategy	Described in the methods section and figure legends.

☒ Tick this box to confirm that a figure exemplifying the gating strategy is provided in the Supplementary Information.
