# Supplementary figures and images for: Somatic deficiency of the human E3 ubiquitin ligase CBL in leukocytes impairs B cell but not T cell development and function
Source: Nat Immunol. 2026 Jan 15;27(2):308–22. doi: 10.1038/s41590-025-02381-7 (PMC12864045; doi:10.1038/s41590-025-02381-7)

Figure 4J:

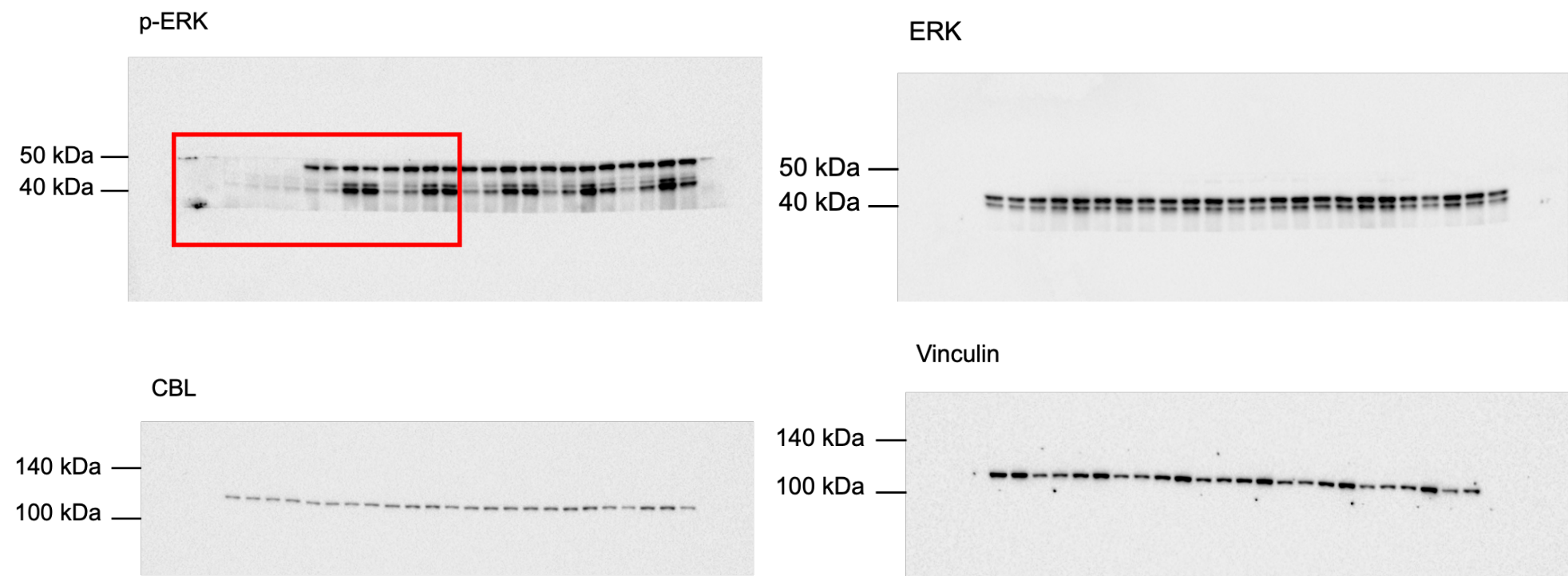

Figure 5D:

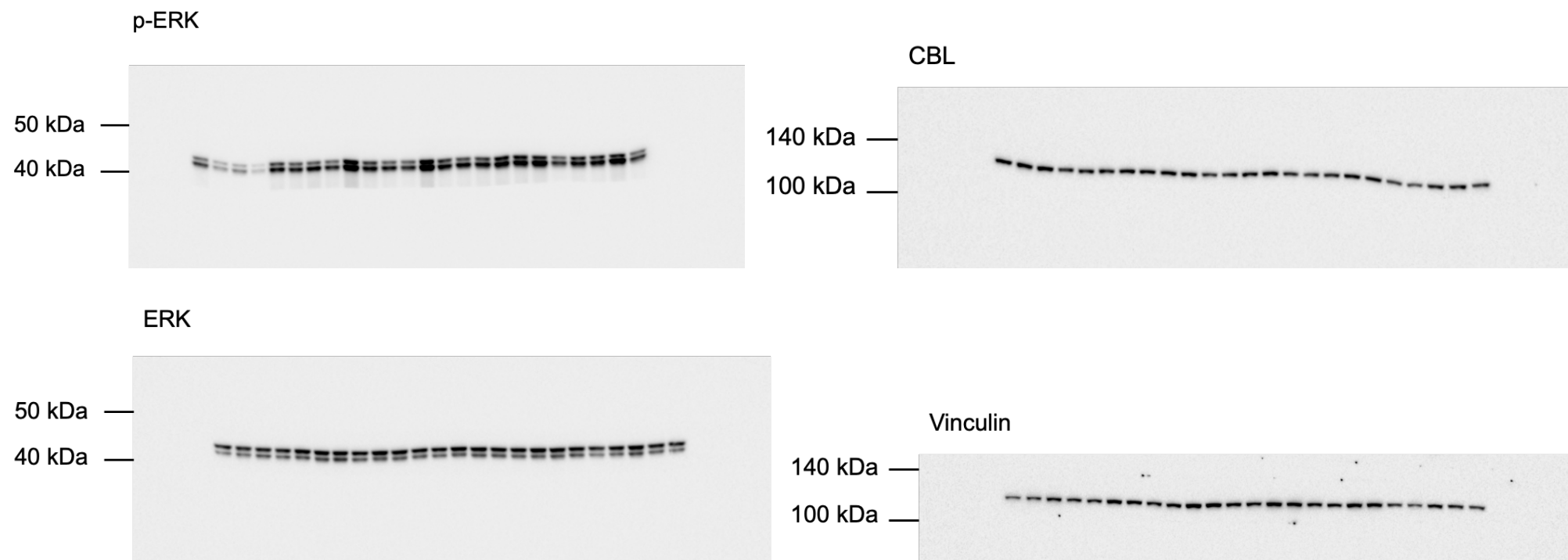

ED Figure 4D:

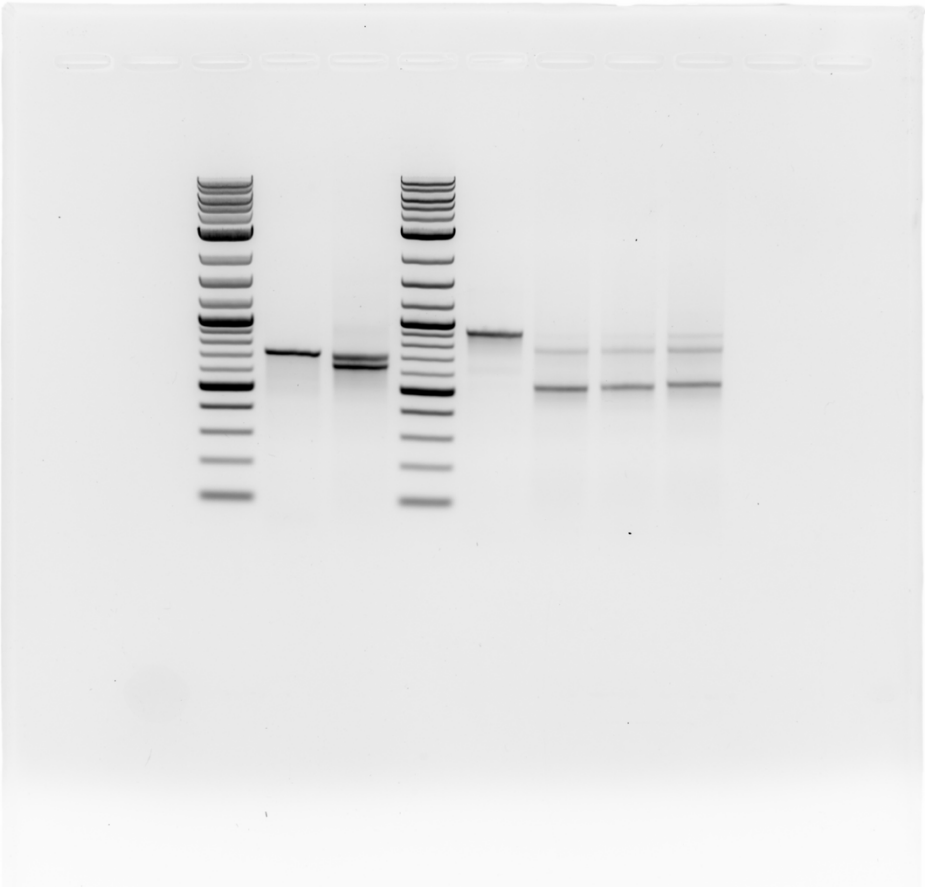

ED Figure 4F:

GAPDH

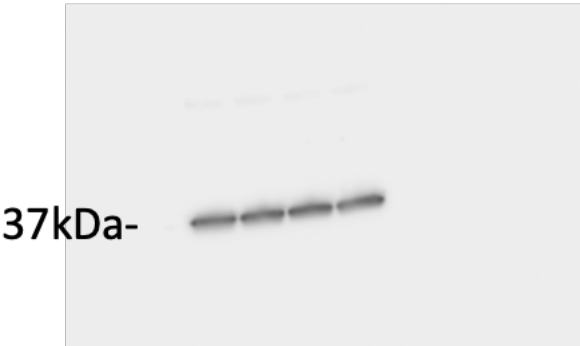

CBL

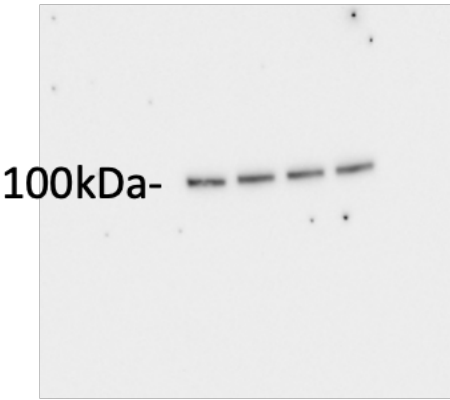

ED Figure 6A:

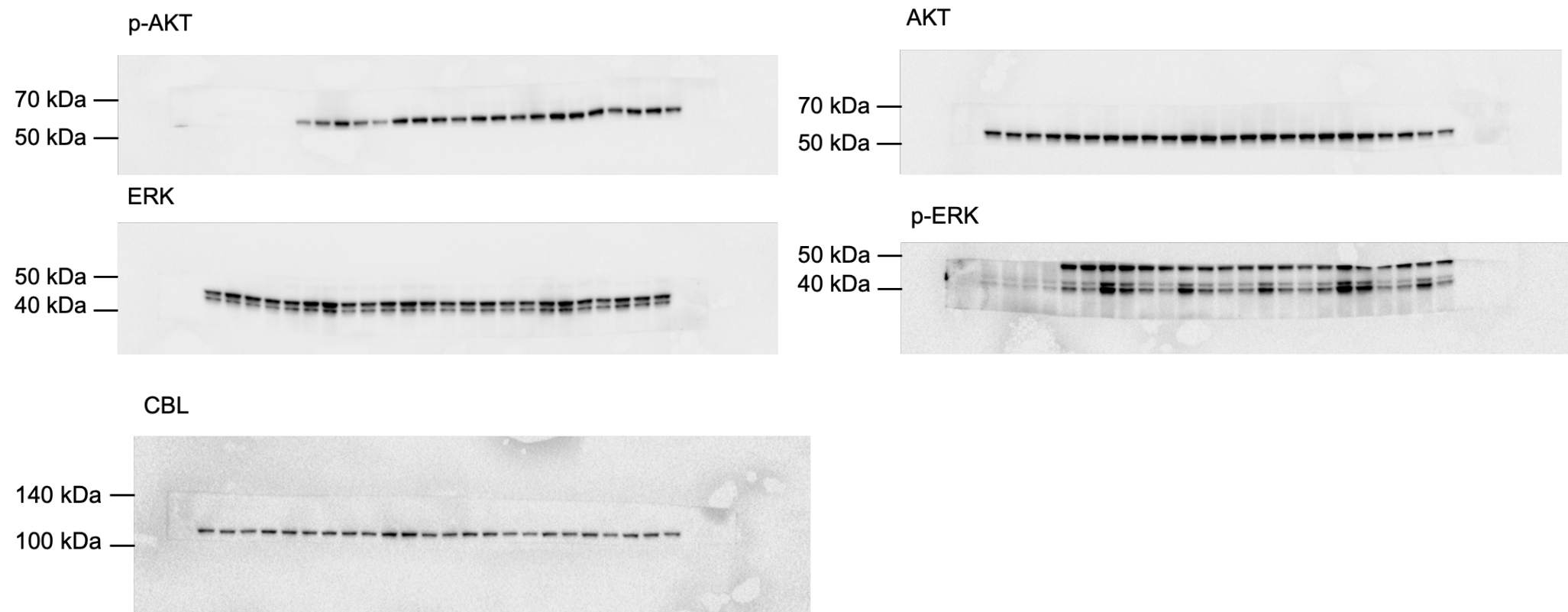

ED Figure 6D:

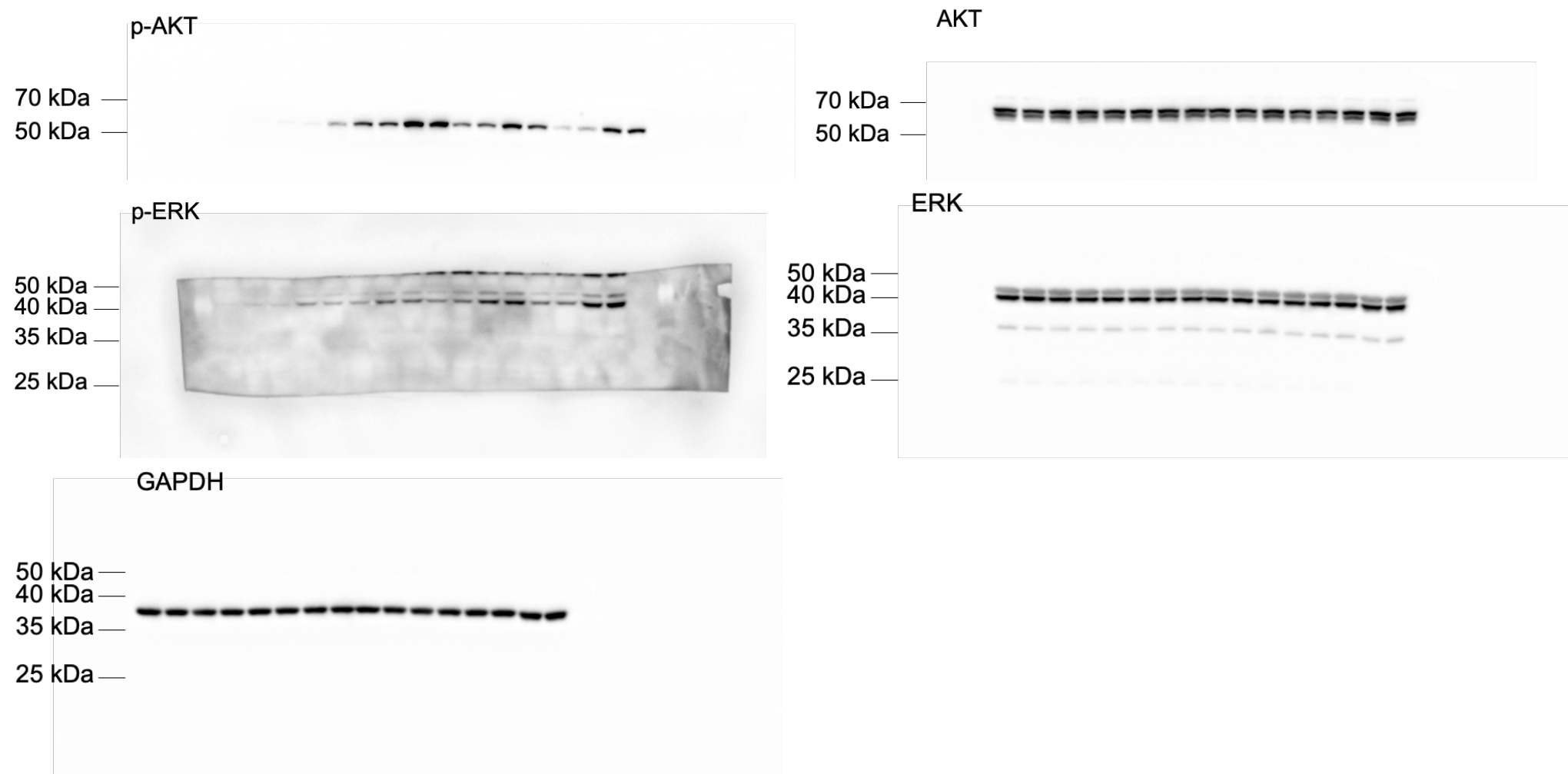

ED Figure 7C:

CBL

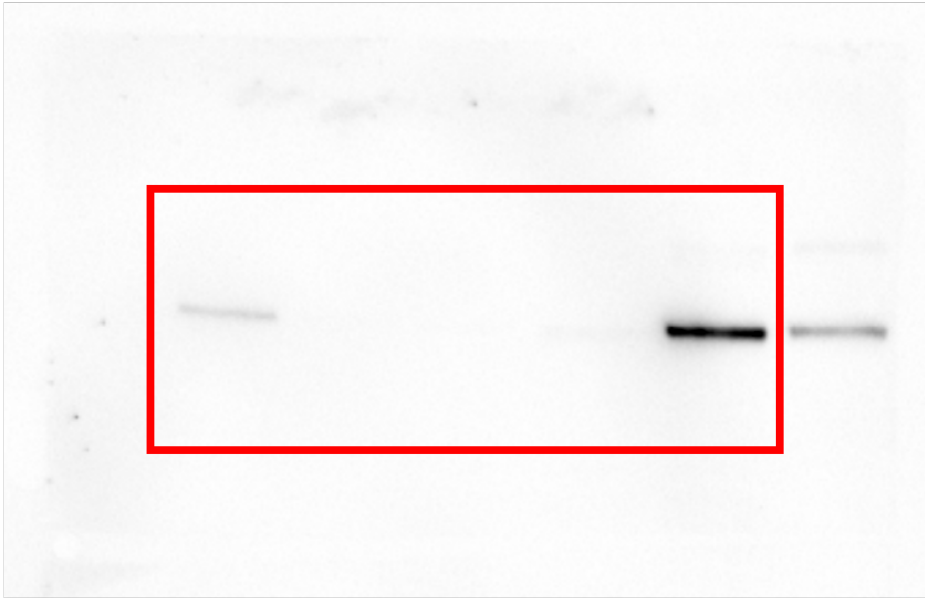

ERK

40kDa-

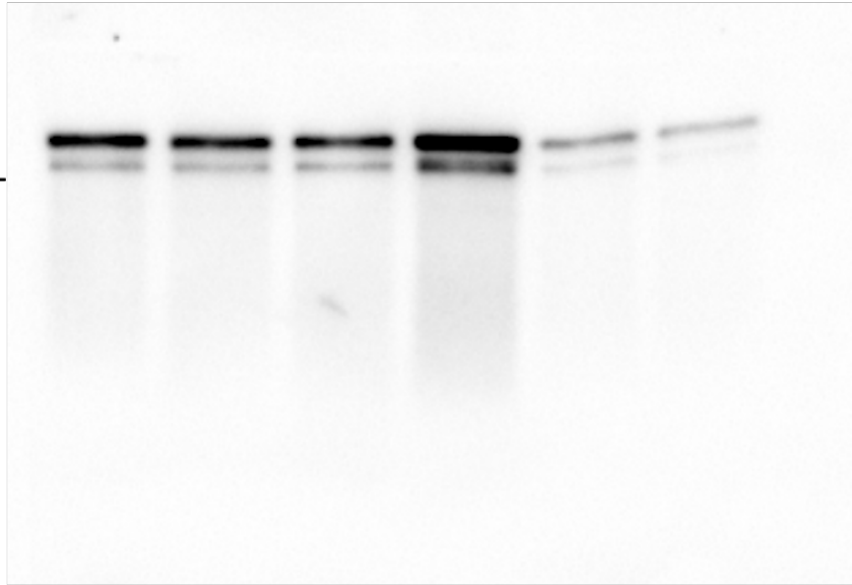

Supplement: Supplementary file 6 — All scans of full membranes and DNA gels. [file 41590_2025_2381_MOESM6_ESM.pdf]
